# Salvianolic Acid B Suppresses ER Stress-Induced NLRP3 Inflammasome and Pyroptosis via the AMPK/FoxO4 and Syndecan-4/Rac1 Signaling Pathways in Human Endothelial Progenitor Cells

**DOI:** 10.1155/2022/8332825

**Published:** 2022-03-17

**Authors:** Yubo Tang, Qingde Wa, Longyun Peng, Yifan Zheng, Jie Chen, Xiao Chen, Xuenong Zou, Huangxuan Shen, Shuai Huang

**Affiliations:** ^1^Department of Pharmacy, The First Affiliated Hospital, Sun Yat-sen University, 510080 Guangzhou, China; ^2^Department of Orthopaedic Surgery, The Second Affiliated Hospital of Zunyi Medical University, 563000 Zunyi, China; ^3^Department of Cardiology, The First Affiliated Hospital, Sun Yat-sen University, 510080 Guangzhou, China; ^4^Guangdong Provincial Key Laboratory of Orthopedics and Traumatology, Department of Spinal Surgery, The First Affiliated Hospital, Sun Yat-sen University, 510080 Guangzhou, China; ^5^State Key Laboratory of Ophthalmology, Zhongshan Ophthalmic Center, Sun Yat-sen University, Guangzhou 510060, China; ^6^Department of Orthopaedic Surgery, The Second Affiliated Hospital, Guangzhou Medical University, 510260 Guangzhou, China

## Abstract

Mounting evidence demonstrates uncontrolled endoplasmic reticulum (ER) stress responses can activate the inflammasome, which generally results in endothelial dysfunction, a major pathogenetic factor of chronic inflammatory diseases such as atherosclerosis. Salvianolic acid B (SalB), produced by Radix Salviae, exerts antioxidative and anti-inflammatory activities in multiple cell types. However, SalB's effects on ER stress-related inflammasome and endothelial dysfunction remain unknown. Here, we showed SalB substantially abrogated ER stress-induced cell death and reduction in capillary tube formation, with declined intracellular reactive oxygen species (ROS) amounts and restored mitochondrial membrane potential (MMP), as well as increased expression of HO-1 and SOD2 in bone marrow-derived endothelial progenitor cells (BM-EPCs). ER stress suppression by CHOP or caspase-4 siRNA transfection attenuated the protective effect of SalB. Additionally, SalB alleviated ER stress-mediated pyroptotic cell death via the suppression of TXNIP/NLRP3 inflammasome, as evidenced by reduced cleavage of caspase-1 and interleukin- (IL-) 1*β* and IL-18 secretion levels. Furthermore, this study provided a mechanistic basis that AMPK/FoxO4/KLF2 and Syndecan-4/Rac1/ATF2 signaling pathway modulation by SalB substantially prevented BM-EPCs damage associated with ER stress by decreasing intracellular ROS amounts and inducing NLRP3-dependent pyroptosis. In summary, our findings identify that ER stress triggered mitochondrial ROS release and NLRP3 generation in BM-EPCs, while SalB inhibits NLRP3 inflammasome-mediated pyroptotic cell death by regulating the AMPK/FoxO4/KLF2 and Syndecan-4/Rac1/ATF2 pathways. The current findings reveal SalB as a potential new candidate for the treatment of atherosclerotic heart disease.

## 1. Introduction

Atherosclerosis (AS), the top etiologic agent of cardiovascular disease (CVD), represents the leading cause of death globally, with elevated morbidity and mortality [1]. The vascular endothelium has attracted increasing attention for its paracrine, endocrine, and autocrine activities and is considered an important factor in vascular homeostasis, rather than a mere barrier separating the blood from the arterial wall [2]. Therefore, the vascular endothelium is regarded as an essential site for a large number of pathophysiological events in multiple cardiometabolic pathologies, particularly AS. Characterized by endothelium dysfunction, foam cell formation, and lymphocyte infiltration, AS is widely considered the pathological result of prolonged and excessive inflammatory processes [3, 4]. It was demonstrated that AS has a close association with innate immunity, featuring pyroptosis, a novel caspase-1-dependent inflammatory type of cell death that was firstly detected in *Salmonella typhimurium*-infected macrophages [5]. Caspase-1 induction requires the generation and activation of the inflammasome, which represents a multiprotein complex playing critical regulatory roles in innate immunity and inflammation. Of all nucleotide-binding oligomerization domain-like receptor (NLR) family members, the NLR family pyrin domain-containing-3 (NLRP3) inflammasome is the most widely investigated, comprising NLRP3, the adaptor apoptosis-associated speck-like (ASC) protein and procaspase-1 [6]. Inflammasomes are multiprotein complexes comprising a sensor protein (e.g., an NLR family member), an adaptor protein ASC encompassing a CARD, and caspase-1 cleaving cytokines. As opposed to other inflammasomes that have specific triggers, the NLRP3 inflammasome can be induced by multiple physical and chemical factors, e.g., adenosine triphosphate (ATP), cholesterol crystal, lipopolysaccharide (LPS), potassium efflux, and mitochondrial dysfunction-derived signals, including reactive oxygen species (ROS), oxidized mtDNA, and externalization of phospholipid cardiolipin [7, 8]. In addition, critical roles for NLRP3 inflammasome, IL-1*β*, IL-18, and pyroptosis have been demonstrated in AS [9].

Factors including oxidative stress, ischemia, and altered calcium homeostasis increase the amounts of unfolded proteins, promoting endoplasmic reticulum (ER) stress. Growing evidence suggests ER stress is involved in the pathogenesis of cardiovascular diseases [10]. ER stress induces the unfolded protein response (UPR), which is involved in the pathogenetic mechanisms of multiple cardiovascular ailments, including heart failure, stroke, and kidney failure. Moreover, ER stress triggers a pathological stress cascade that causes oxidative stress, inflammation, and apoptosis and is involved in endothelial dysfunction [11, 12]. ER stress-associated oxidative stress and inflammatory response are currently considered major players in the pathogenetic mechanisms of metabolic diseases, including diabetes mellitus, obesity, and AS [13, 14]. Recently, it was shown that ER stress causes the dissociation of TXNIP from TRX and then activates the NLRP3 inflammasome, which regulates inflammatory reactions and cell death via caspase-1-dependent processing of mature IL-1*β* [15]. Activated caspase-1, meanwhile, can promote caspase-1-dependent pyroptosis [16]. Therefore, suppressing ER stress-mediated NLRP3 inflammasome induction could significantly attenuate pyroptosis and IL-1*β* release, thereby alleviating AS.

Salvianolic acid B (SalB), produced by Radix Salvia miltiorrhiza (Danshen), has a wide range of therapeutic effects, including antioxidative, antitumor, anti-inflammatory, and antiatherosclerotic properties. SalB has pronounced cardioprotective activity by increasing cell survival, suppressing apoptosis, and preserving normal cell functions and possesses the capability of protecting from doxorubicin-induced cardiac dysfunction by suppressing ER stress-mediated cardiomyocyte apoptosis through the inhibition of Ca^2+^ overload [17, 18]. However, how SalB inhibits ER stress is largely unknown. Therefore, deciphering the exact mechanism by which the delicate balance of ROS generation and neutralization is maintained in vascular wall cells is critical for the development of anti-ER stress agents with high efficiency.

This work firstly showed that ER stress-induced ROS generation, NLRP3 inflammasome induction, and subsequent pyroptosis in bone marrow-derived endothelial progenitor cells (BM-EPCs) could be attenuated by SalB treatment. In addition, we demonstrated that SalB exerts antipyroptotic effects mediated by ER stress in BM-EPCs by modulating the AMPK/FoxO4/KLF2 and Syndecan-4/Rac1/ATF2 pathways.

## 2. Materials and Methods

### 2.1. Reagents

Tauroursodeoxycholic acid (TUDCA, purity ≥ 95%), Compound C, AICAR, 2′,7′-dichlorofluorescin diacetate (DCFH-DA, purity ≥ 97%), and z-YVAD-FMK (purity ≥ 95%) were obtained from Sigma Aldrich. Rainbow markers were purchased from GE Healthcare. AICAR was provided by Selleck Chemicals. Tunicamycin (TM, purity ≥ 98%), monoclonal antibodies specific to HO-1, SOD2, CHOP, GRP78, ATF4, p-eIF2*α*, eIF2*α*, TXNIP, caspase-4, Bcl-xL, Bax, caspase-1 (p20), cleaved caspase-3, cytochrome *c*, p-AMPK*α*, AMPK*α*, p-FoxO4, FoxO4, KLF2, Syndecan-4, Rac1, p-ATF2, ATF2, NLRP3, ASC, IL-1*β*, GAPDH, *α*-tubulin, and horseradish peroxidase-conjugated anti-rabbit antibodies were provided by Cell Signaling Technologies.

### 2.2. Cell culture and SalB treatment

Bone marrow specimens were collected from 12 healthy individuals (29-62 years of age, averaging 46.5 years) after signed informed consent. The study was approved by the local institutional review board (no. 2016[130]). Human BM-EPCs were isolated, cultured, and identified as reported previously [19]. SalB (purity ≥ 99%; TautoBiotec, Shanghai, China) was applied in in vitro assays. A stock solution of SalB (100 mM) was made by dissolution in DMSO, and further dilution used a serum-free culture medium.

### 2.3. Analysis of Cell Viability

BM-EPCs were administered with SalB for 24 h, 48 h, and 72 h, respectively, followed by incubation with 5 *μ*g/mL TM for 24 h for ER stress damage induction. A MTS assay kit (CellTiter 96 Aqueous One Solution Cell Proliferation Assay) from Promega (USA) was utilized for cell viability assessment as directed by the manufacturer. Optical density was read at 490 nm on a spectrophotometer. Triplicates were used for each experimental condition.

### 2.4. LDH Release Assay

LDH is released by dead cells, and the released amounts were assessed in this work with a Promega kit as directed by the manufacturer. Briefly, BM-EPCs administered with SalB for 48 h were incubated with 5 *μ*g/mL TM for 24 h to induce ER stress injury. Then 50 *μ*L each of cell culture supernatant and 50 *μ*L substrate were mixed and incubated at ambient for 30 min. After stopping the reaction, optical density was read at 492 nm. LDH release was derived as follows: Percentage of cytotoxicity = (Absorbance of the experimental sample/Absorbance of maximun LDH release) × 100%.

### 2.5. Assessment of Cell Survival (Live-Dead Assay)

Cell survival was assessed with a calcein-AM/ethidium homodimer-1 (EthD-1) dual-staining assay kit (Molecular Probes, USA), as directed by the manufacturer. Briefly, BM-EPCs administered with SalB for 48 h were incubated with 5 *μ*g/mL TM for 24 h. After rinsing with PBS, cells were supplemented with 2 *μ*M calcein-AM and 4 *μ*M EthD-1 in 100 *μ*L PBS for 30 min at 37°C. A fluorescence microscope (Leica, DMi8, Wetzlar, Germany) was utilized for analysis.

### 2.6. Apoptosis Assessment

An Annexin V-FITC/PI apoptosis detection kit (Miltenyi, Germany) was utilized to evaluate apoptosis as directed by the manufacturer. Briefly, BM-EPCs administered with SalB for 48 h were incubated with 5 *μ*g/mL TM for 24 h. Then, cells were harvested by trypsinization and exposed to Annexin V-FITC and PI at ambient for 10 min shielded from light. An LSRII system (BD™, Germany) was utilized for flow cytometry analysis. FlowJo (Tree Star, USA) was used for quantitation.

### 2.7. Caspase Activity Assay

BM-EPCs administered with 10 or 20 *μ*M SalB for 48 h underwent incubation with TM for 24 h. The harvested cells were resuspended in lysis buffer and kept on ice for 1 h. After clearing the lysates by centrifugation at 4°C (12,000 g, 30 min), they were added to a 96-well plate containing substrates specific for caspase-8 (Ac-IETD-AFC), caspase-3 (Ac-DEVD-AMC), caspase-4 (Ac-LEVD-AF), and caspase-1 (Ac-YVAD-AFC) at 37°C for 1 h. Caspase activity was assessed on a fluorimeter (excitation and emission at 380 and 440 nm for AMC, respectively, and 400 and 500 nm for AFC, respectively).

### 2.8. Migration Assay

Cell migration was assessed utilizing the Transwell system with 8 *μ*m pores (Corning, USA). Totally 5 × 10^4^ cells in serum-free medium were added to the upper compartment, while the lower one was added with 600 *μ*L EGM-2 containing 5% FBS. Following 48 h incubation, cells on the lower side of the membrane underwent fixation and staining with Alexa Fluor 488® phalloidin for 30 min. Analysis was performed with a Leica DMi8 inverted fluorescence microscope (Leica). Triplicate assays were carried out, and cells were counted in 10 randomly selected high-power fields.

### 2.9. Capillary Tube Formation Assay

This was carried out with the ECMatrix™ (BD Biosciences), as directed by the manufacturer. Totally 1 × 10^4^ cells were used per well, and an inverted light microscope (Zeiss Axio Observer Z1, Germany) was utilized for analysis. Five distinct high-power fields were examined per sample for the number of tubes/fields.

### 2.10. ROS Level Assessment

ROS amounts in BM-EPCs were assessed with 2,7-dichlorodihydro-fluorescindiacetate (DCFH-DA). Treated cells underwent incubation with DCFH-DA (10 *μ*M) for 30 min at 37°C and three PBS washes. Then, 2′,7′-dichlorofluorescin (DCF) fluorescent signals were assessed on a fluorescence microplate reader (excitation/emission at 488/520 nm).

### 2.11. Immunofluorescence Labeling

BM-EPCs were cultured on poly-L-lysine coverslips and underwent 4% formalin fixation for 20 min at ambient, followed by permeabilization with 0.3% TritonX-100 at ambient for 20 min. Samples blocked with 5% BSA for 1 h underwent incubation with primary antibodies in PBS supplemented with 1% BSA and 0.1% Tween-20 overnight at 37°C. The samples then underwent further incubation with Alexa Fluor-linked fluorescent secondary antibodies diluted at 1 : 2000 in PBS for 1 h. The nuclei in the samples were stained with Hoechst 33342 diluted at 1 : 20000 in PBS for 15 min at ambient. Finally, the coverslips containing the samples underwent mounting on glass slides with the Gold antifade reagent (Invitrogen, USA).

### 2.12. Cytochrome *c* Detection in Cytoplasmic Fractions

Cell lysis was performed with 200 *μ*L of lysis buffer (20 mM HEPES–KOH (pH 7.4), 50 mM KCl, 70 mM sucrose, 2 mM MgCl_2_, and 1 mM EDTA) supplemented with 0.1 mM PMSF and protease inhibitor cocktail (Sigma). After harvest by scraping, treated cells underwent homogenization on ice by 10 passages through a 21G needle. The obtained homogenates then underwent centrifugation (10,000 g for 15 min; 4°C), and the supernatants were cleared by another centrifugation (17,000 g for 30 min; 4°C). The resulting supernatants were used as protein samples for SDS-PAGE and immunoblot for cytochrome *c* detection. Poly (ADP-ribose) polymerase (PARP) was used as a control for ensuring the absence of nuclear proteins.

### 2.13. Cytokine Assays

ELISA kits were employed for assessing secreted IL-1*β* (Thermo fisher, BMS224-2TEN) and IL-18 in cell culture supernatants after 24 h of treatment, as directed by the manufacturer.

### 2.14. Transfection with Small Interfering RNAs (siRNAs)

BM-EPCs seeded in 35 mm plates at 5 × 10^5^/dish underwent culture in antibiotic-free medium overnight. This was followed by transfection for 48 h with siRNAs targeting CHOP, caspase-4, AMPK, FoxO4, Syndecan-4, Rac1, and NLRP3, respectively, or a scrambled control siRNA, with the Lipofectamine RNAi Max reagent (Invitrogen) as directed by the manufacturer. Quantitative real-time PCR (qRT-PCR) and immunoblot were performed to confirm silencing.

### 2.15. RNA Extraction and Quantitative Real-Time PCR

Total RNA isolation used the RNeasy Mini Kit (QIAGEN, Germany). Template RNA (30 *μ*g) was used to synthesize the first-strand cDNA with a reverse transcription kit (Takara, China). The mRNA amounts were quantified on a Bio-RadiQ5 Gradient Real-Time PCR system (Bio-Rad, USA), with GAPDH as a reference gene. Triplicate assays were repeated five times.

### 2.16. Immunoblot

40 *μ*g of total protein underwent separation by 10% SDS-PAGE and electrotransfer onto a polyvinylidene difluoride (PVDF) membrane. The membrane underwent blocking with 5% skimmed milk and successive incubations with primary (overnight at 4°C) and secondary (1 h at ambient) antibodies. Enhanced chemiluminescence (GE, USA) was utilized for detection. Quantity One 4.6.2 (Bio-Rad) was utilized for quantitation. At least three assays were performed independently.

### 2.17. Statistical Analysis

Continuous variables are mean ± standard deviation from three or more assays, unless otherwise stated. SPSS 16.0 was utilized for data analysis. Independent-sample *t*-test and one-way ANOVA with post hoc Tukey's test were carried out for group pair and multiple group comparisons, respectively. *P* < 0.05 was deemed to indicate statistical significance.

## 3. Results

### 3.1. SalB Prevents TM-Dependent Cell Death in BM-EPCs by Modulating Apoptosis

**Figure 1:**
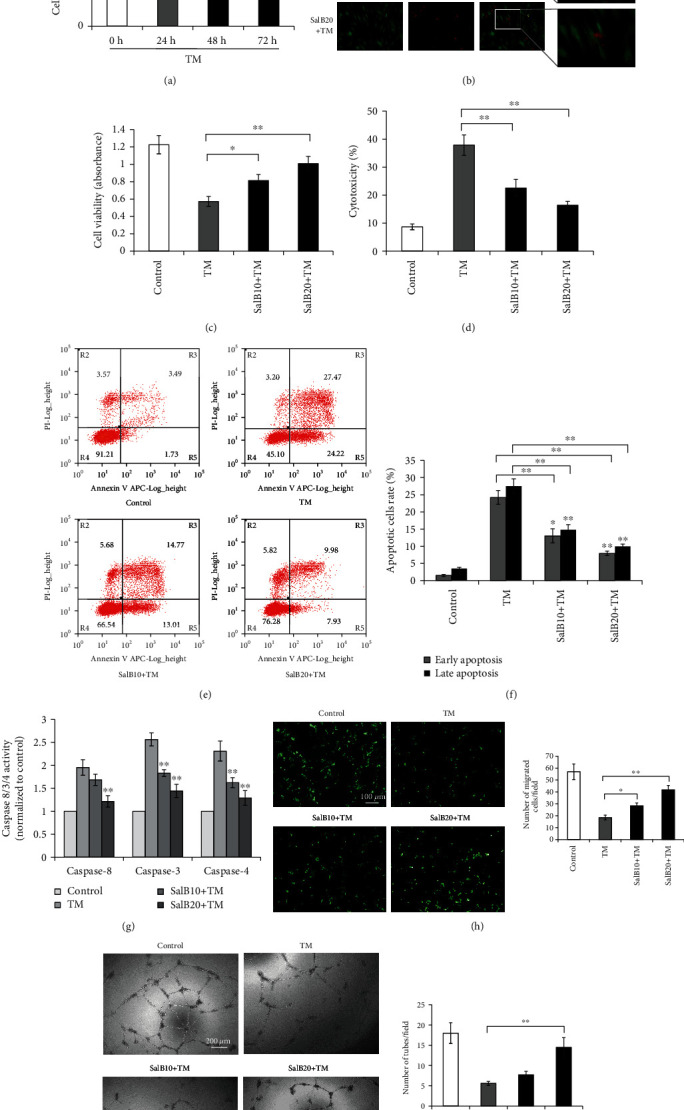
SalB protects BM-EPCs from TM-associated cell death by modulating apoptosis. (a) BM-EPCs were administered 5 *μ*g/mL TM for 24, 48, or 72 h and examined by the MTS assay, with nontreated cells as the negative control group, *n* = 6. (b) BM-EPCs were administered 0, 10, or 20 *μ*M SalB for 48 h and incubated with 5 *μ*g/mL TM for 24 h. Cell viability was assessed by calcein-AM/EtD-1 double staining. Representative photographs are shown. (c, d) BM-EPCs underwent the treatments described in (b), followed by the MTS assay (cell viability/proliferation, *n* = 5) and LDH assay (cell death, *n* = 6). (e–g) Cells underwent the treatments described in (b), followed by harvesting and labeling with Annexin V-FITC and PI; determination of apoptosis used flow cytometry (*n* = 7). Caspase assay was carried out for measuring the activities of caspases-8, caspases-3, and caspases-4 (*n* = 5). (h, i) Cells underwent the treatments described in (b), and cell migration was assessed by the Transwell assay (cells in serum-free medium and medium with 10% FBS in the upper and lower chambers, respectively). Following 4 h incubation, cells on the upper side of the membrane were removed, and the membrane underwent staining with Alexa Fluor 488®. A fluorescence microscope was utilized to image migrating cells in 10 random high-power fields (×100, *n* = 5). Upon cell culture on Matrigel™ for 18 h under normal growth conditions, capillary tube formation was examined by inverted light microscopy in 5 high-power fields (×100, *n* = 6). Data are mean ± SD, ^∗^*P* < 0.05, ^∗∗^*P* < 0.01 versus the 0 h or control group.

ER stress triggered by TM reduced BM-EPC amounts as early as 24 h, to a similar degree compared to the 48 and 72 h time points (Figure 1(a)). To assess the cardioprotective effects of SalB on ER stress-dependent cytotoxicity, BM-EPCs were preadministered with SalB for 48 h before TM administration. As shown in Figure 1(b), while without TM almost the totality of cells was viable, administration of 5 *μ*g/mL TM resulted in substantial cell injury, with shrinking and nuclear membrane wrinkling. Meanwhile, preincubation with SalB resulted in markedly reduced amounts of EthD-1-positive cells; in this group, the cells were morphologically intact with no sign of cytoplasmic damage. The rate of viable cells after administration of 5 *μ*g/mL TM for 24 h was decreased to 46.75% versus control cells. Notably, these values were markedly increased to 66.47% and 82.32% after pretreatment with 10 and 20 *μ*M SalB, respectively (Figure 1(c)). SalB-related protection was confirmed by the LDH assay (Figure 1(d)) (*P* < 0.01). The above findings suggested that SalB protects BM-EPCs from ER stress-dependent cell injury.

To assess whether cell apoptosis is involved in TM cytotoxicity, apoptosis was measured by the Annexin V-FITC/PI double staining assay. Early apoptotic (Annexin V-positive and PI-negative) cells showed markedly reduced rates in the SalB groups, from 27.47% to 14.77% and 9.98% in cells pretreated with SalB at 10 and 20 *μ*M for 48 h, respectively (Figures 1(e) and 1(f)) (*P* < 0.01). Since caspases are important in apoptosis, their activation (cleavage) was next examined. Exposure to TM markedly enhanced caspase-8, caspase-3, and caspase-4 activities, and considerable decreases in caspase activities were found upon SalB pretreatment for 48 h (Figure 1(g)).

To assess the possible effects of TM on BM-EPC functions, cell migration and capillary tube formation assays were carried out. As shown in Figure 1(h), migration in TM-treated cells was remarkably decreased compared with control cells, and 10 and 20 *μ*M SalB remarkably increased the amounts of migrating cells by 1.53 ± 0.21-fold and 2.26 ± 0.34-fold in comparison with TM-treated cells, respectively (*P* < 0.01) (Figure 1(h)). In addition, the inhibition of sprouting tubules by TM was dramatically attenuated after preincubation with SalB by 11.67 ± 2.11% (10 *μ*M) and 44.82 ± 10.4% (20 *μ*M) compared with TM-treated BM-EPCs (Figure 1(i)).

### 3.2. ROS Suppression by SalB Attenuates TM-Induced Cell Death in BM-EPCs

**Figure 2:**
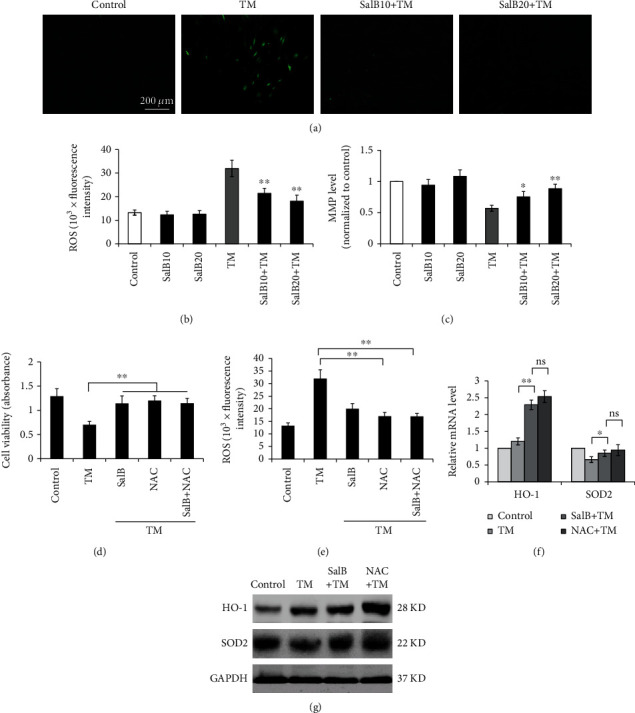
ROS suppression by SalB attenuates TM-induced cell death in BM-EPCs. (a, b) Cells were first incubated with 10 and 20 *μ*M SalB for 48 h prior to TM administration at 5 *μ*g/mL for 24 h. This was followed by incubation with H_2_DCFDA (30 *μ*M) for fluorescence microscopy. ROS amounts were reflected by cell DCF production. Fluorescence quantitation used a fluorescence microplate reader (*n* = 6). (c) BM-EPCs underwent the treatments described in (a) and incubation with rhodamine 123 for 20 min shielded from light. After washing with PBS, fluorescence measurement was carried out on a fluorescence plate reader (*n* = 6). (d, e) BM-EPCs were pretreated with SalB or the ROS inhibitor NAC, followed by stimulation with TM for 24 h. The MTS assay was performed to determine cell viability, and ROS amounts were reflected by cell DCF levels, assessed on a fluorescence microplate reader (*n* = 7). (f, g) BM-EPCs underwent the treatments described in (d, e), and the gene expression levels of HO-1 and SOD-2 were assessed by qRT-PCR; with GAPDH as a reference gene, the 2^−*ΔΔ*Ct^ method was utilized for analysis (*n* = 5). Cell lysates were obtained for immunoblot. Data are mean ± SD, ^∗^*P* < 0.05, ^∗∗^*P* < 0.01 versus the control or indicated group.

ROS are synthesized by damaged mitochondria and could trigger inflammasome activation [20]. As shown in Figures 2(a) and 2(b), after exposure to 5 *μ*g/mL TM for 24 h, markedly elevated ROS production was found in BM-EPCs (241.7% ± 11.0% TM group vs. control cells), while SalB preincubation decreased ROS generation to significantly lower amounts (162.0% ± 9.9% and 136.7% ± 13.9% in the 10 and 20 *μ*M SalB groups, respectively; *P* < 0.01), indicating SalB exerted powerful ROS scavenging effects. Moreover, TM at 24 h induced MMP dissipation, reflected by reduced green fluorescence. Preincubation with 10 and 20 *μ*M SalB for 48 h, respectively, before TM administration alleviated MMP dissipation, with enhanced fluorescent signals, almost reaching control values (Figure 2(c)). However, SalB administered alone showed no marked effects on basal ROS and MMP levels (Figures 2(b) and 2(c)). Additionally, N-acetyl-L-cysteine (NAC) administration markedly alleviated TM-dependent ROS production and cytotoxicity and abolished the attenuating effects of SalB in response to TM (Figures 2(d) and 2(e)).

Mitochondria have an antioxidant enzymatic system for protection against extreme oxidative stress. HO-1 represents a protein with an enzymatic function that has cytoprotective effects such as anti-inflammatory and antiapoptotic properties [21]. Superoxide dismutase 2 (SOD2) is a major antioxidant enzyme that protects cells against oxidative stress injury by neutralizing ROS. Interestingly, the mRNA and protein levels of HO-1 were significantly elevated in cells incubated with TM and SalB, which were not affected by NAC treatment (Figures 2(f) and 2(g)). However, the mRNA and protein levels of SOD2 were both reduced upon TM exposure and returned to normal in SalB pretreated cells (Figures 2(f) and 2(g)).

### 3.3. SalB Suppresses TM-Dependent Toxicity by Regulating ER Stress-Related Proteins in BM-EPCs

**Figure 3:**
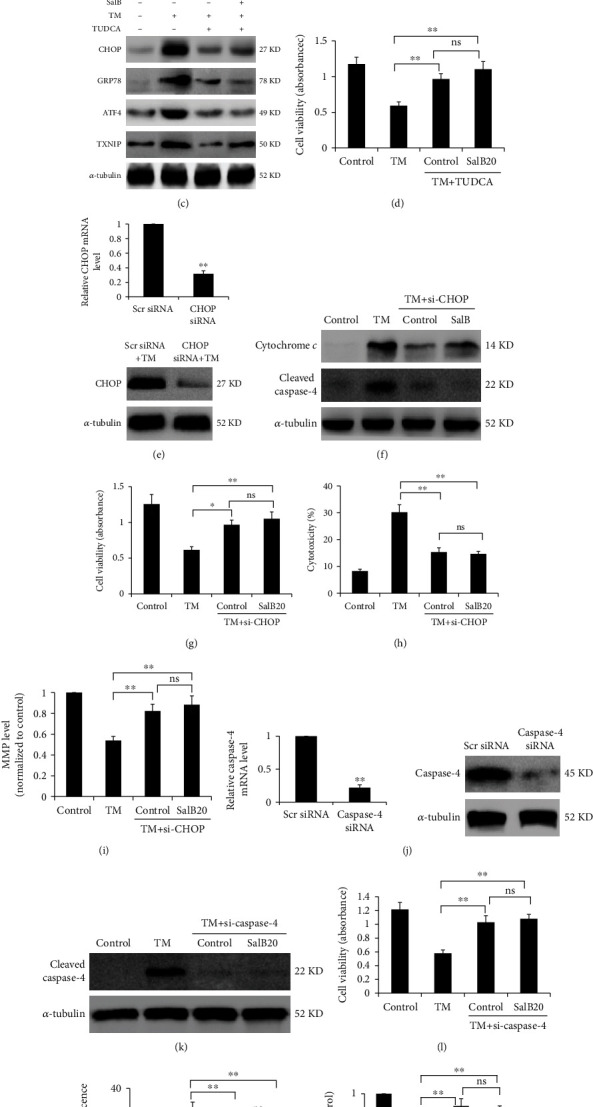
SalB suppresses TM-induced cell death by modulating ER stress-related proteins in BM-EPCs. (a) BM-EPCs were administered TM at 5 *μ*g/mL for 24, 48, or 72 h followed by Western blot for CHOP protein quantitation, with untreated cells as the negative control group. (b) Cells underwent incubation with 0, 10, or 20 *μ*M SalB for 48 h and were administered with TM at 5 *μ*g/mL for 24 h. Cell lysates were obtained for immunoblot. (c, d) BM-EPCs underwent pretreatment with SalB or an ER stress inhibitor (TUDCA), followed by stimulation with TM for 24 h. The MTS assay was performed to determine cell viability (*n* = 6), and the indicated proteins were examined by immunoblot. (e) BM-EPCs underwent transfection with CHOP-specific and scramble siRNAs, respectively, for 48 h, and mRNA and protein amounts were determined for confirming silencing efficiency. (f) BM-EPCs underwent incubation with or without CHOP siRNA for 48 h, followed by treatment with SalB (or vehicle control) and TM stimulation; protein amounts were assessed by Western blot. (g–i) Cell viability, cytotoxicity, and MMP levels were assessed by the MTS assay (*n* = 8), LDH assay (*n* = 5), and rhodamine 123 labeling (*n* = 5), respectively, underwent incubation with or without CHOP siRNA, and exposure to SalB or TM. (j) BM-EPCs underwent transfection with scramble or caspase-4 siRNA for 48 h, followed by administration of SalB (or not) and TM; protein amounts were assessed by immunoblot. (k) BM-EPCs underwent transfection with or without caspase-4 siRNA for 48 h, followed by administration of SalB (or not) and TM; protein amounts were assessed by immunoblot. (l–n) Cell viability, ROS generation, and MMP levels were assessed (*n* = 8); DNA content (*n* = 6) was assessed after incubation with or without caspase-4 siRNA under SalB or TM stimulation. Data are mean ± SD, ^∗^*P* < 0.05, ^∗∗^*P* < 0.01 versus the indicated group. ns: not significant.

For ensuring the pharmacological effects of TM in our setting, we demonstrated that TM at different time points increased the expression of CHOP (Figure 3(a)), which is considered a marker of ER stress and an integral component of ER stress-mediated apoptosis. Moreover, TM administration upregulated GRP78 and ATF4 and increased the phosphorylation of eIF-2*α*; these effects were markedly suppressed by SalB preincubation (Figure 3(b)). Furthermore, administration of TUDCA, a powerful ER stress suppressor, decreased TM-related CHOP, GRP78, and ATF4 activation and downregulated TXNIP, which is an important molecule linking ER stress, inflammation, and apoptosis (Figure 3(c)). In subsequent experiments, TUDCA restored the TM-dependent reduction of cell viability and abolished the attenuating effect of SalB on TM-associated cytotoxicity (Figure 3(d)). Next, siRNA was utilized for CHOP silencing in BM-EPCs, and RT-PCR and immunoblot were carried out to confirm CHOP knockdown (Figure 3(e)). We found that cytochrome *c* and cleaved caspase-4 induced by TM were suppressed in CHOP siRNA-transfected cells (Figure 3(f)). In addition, cell viability showed a significant increase while LDH release was markedly decreased after CHOP silencing; protection by SalB was remarkably enhanced upon CHOP silencing (Figures 3(g) and 3(h)). Moreover, CHOP silencing starkly increased MMP upon TM induction (Figure 3(i)). To assess caspase-4's function in SalB-associated cryoprotection, caspase-4 was silenced in BM-EPCs by the siRNA technology and confirmed by RT-PCR and immunoblot (Figure 3(j)). Caspase-4 silencing starkly alleviated TM cytotoxicity and decreased cleaved caspase-4 amounts in comparison with the scrambled siRNA group (Figures 3(k) and 3(l)). Additionally, caspase-4 silencing decreased ROS amounts and enhanced MMP upon TM-induced ER stress in BM-EPCs (Figures 3(m) and 3(n)), suggesting loss of caspase-4 protects from TM toxicity in BM-EPCs.

### 3.4. SalB Inhibits ER Stress-Induced Cell Death by Suppressing the AMPK/FoxO4/KLF2 Signaling Pathway

**Figure 4:**
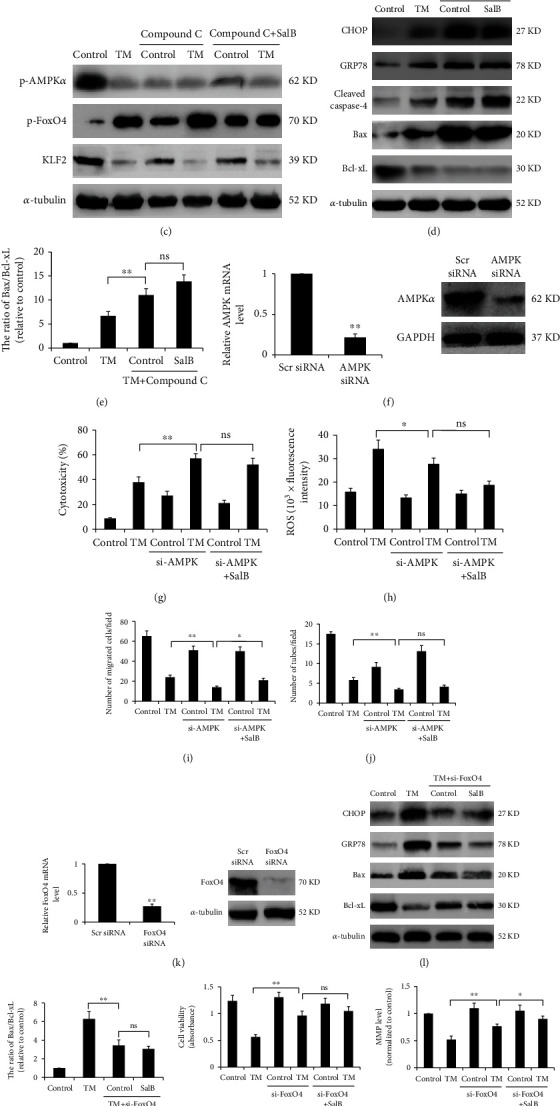
SalB inhibits ER stress-induced apoptosis by suppressing the AMPK/FoxO4/KLF2 signaling pathway. (a, b) Cells were administered 0 or 20 *μ*M SalB for 48 h and incubated with 5 *μ*g/mL TM for 24 h. Anti-p-AMPK*α*, and anti-p-FoxO4 primary antibodies were added to samples, respectively, followed by incubation with secondary DyLight® 594-linked antibodies. DAPI counterstaining was carried out, and a fluorescence microscope was utilized for analysis. (c–e) Totally 48 h following SalB administration, BM-EPCs underwent further incubation with or without the AMPK inhibitor Compound C (10 *μ*M) and TM, and protein amounts were examined by immunoblot. (f, k) BM-EPCs underwent transfection with AMPK or FoxO4-specific and nonspecific siRNAs, respectively, for 48 h, and mRNA and protein amounts were quantitated for assessing silencing efficiency. (g, h) Cytotoxicity and MMP were determined by LDH assay (*n* = 7), and rhodamine 123 labeling (*n* = 5), respectively, after transfection with or without AMPK siRNA and incubation with SalB or TM. (i, j) Cells underwent the treatments described in (c–e), and cell migration was assessed by the Transwell assay (*n* = 7). Capillary tube formation was examined by growing cells on Matrigel™ for 24 h, and average amounts of tubes per field were obtained (*n* = 6). (l, m) BM-EPCs underwent incubation with or without FoxO4 siRNA for 48 h, followed by treatment with SalB (or not) and TM; protein amounts were assessed by immunoblot. (n, o) Cell viability and MMP were assessed by the MTS assay (*n* = 5), and rhodamine 123 labeling (*n* = 6), respectively, after incubation with or without FoxO4 siRNA, and SalB administration or TM stimulation. (p, q) BM-EPCs underwent the treatments described in (l, m), and cell migration was assessed by the Transwell assay (*n* = 7). Capillary tube formation was assessed by growing cells on Matrigel™ for 24 h, and average amounts of tubes per field were assessed (*n* = 5). Data are mean ± SD, ^∗^*P* < 0.05, ^∗∗^*P* < 0.01 versus the indicated group. ns: not significant.

AMPK is known to contribute to ER stress-induced cell injury [22]. Consequently, whether SalB modulates AMPK was examined by immunoblot in BM-EPCs. Figures 4(a) and 4(b) show that TM induction almost blunted p-AMPK*α* and KLF2 expression, which was restored by SalB (20 *μ*M). Notably, TM treatment was associated with markedly increased amounts of phosphorylated FoxO4, which was downregulated by SalB. We hypothesized that AMPK/FoxO4/KLF2 signaling drives the detected cell survival enhancement after ER stress induction. Therefore, inhibition assays were carried out with Compound C. As depicted in Figure 4(c), Compound C reversed SalB-mediated AMPK*α* and FoxO4 phosphorylation in response to TM, as well as KLF2 expression. In addition, CHOP, GRP78, and cleaved caspase-4 and caspase-3 amounts induced by TM were enhanced in cells pretreated with Compound C, and the Bax/Bcl − xL ratio was significantly heightened (Figures 4(d) and 4(e)). To confirm AMPK's contribution to SalB-associated cytoprotection, AMPK was silenced and confirmed by RT-PCR and immunoblot (Figure 4(f)). More importantly, cell viability and MMP were remarkably decreased while LDH levels were markedly elevated after AMPK knockdown; SalB-related protection was markedly decreased after AMPK downregulation (Figures 4(g) and 4(h)). Moreover, suppression of AMPK in BM-EPCs resulted in a significantly reduced number of migrating cells and tubes formed on Matrigel (Figures 4(i) and 4(j)). These results implied that AMPK is critical to angiogenesis.

To further examine FoxO4's role in SalB-induced protection, the siRNA technology was utilized for FoxO4 silencing in BM-EPCs (Figure 4(k)). As shown in Figures 4(l) and 4(m), knockdown of FoxO4 promoted the inhibitory effects of SalB on ER stress-associated proteins, including CHOP and GRP78, and blunted the attenuating effects of SalB on the Bax/Bcl − xL ratio. Moreover, cells with lower FoxO4 expression exhibited higher cell viability and restored MMP after TM treatment in comparison with control cells (Figures 4(n) and 4(o)). Furthermore, suppression of FoxO4 starkly elevated cell migration and tube formation (Figures 4(p) and 4(q)). Taken together, the above findings suggested SalB exerted cytoprotective effects through AMPK/FoxO4/KLF2 signaling.

### 3.5. SalB Inhibits TM-Induced Cell Death by Modulating Syndecan-4/Rac1/ATF-2 Signaling in BM-EPCs

**Figure 5:**
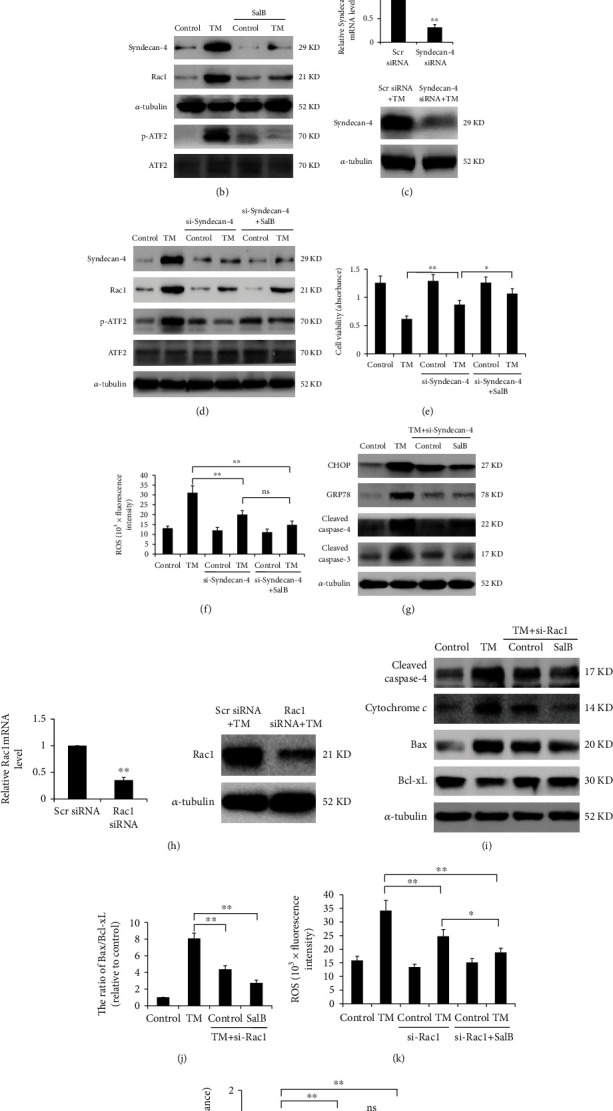
SalB inhibits TM-induced cell death by modulating Syndecan-4/Rac1/ATF-2 signaling in BM-EPCs. (a) Cells were administered 0 or 20 *μ*M SalB for 48 h and incubated with TM at 5 *μ*g/mL for 24 h. Anti-Syndecan-4 primary antibodies were added to samples, with subsequent incubation with secondary DyLight® 594-linked antibodies. DAPI counterstaining was followed by analysis under a fluorescence microscope. (b) BM-EPCs underwent the treatments described in (a), and the indicated proteins were detected by immunoblot. (c) BM-EPCs underwent transfection with Syndecan-4 or scramble siRNA or 48 h; mRNA and protein amounts were obtained for determining silencing efficiency. (d–f) BM-EPCs underwent incubation with or without Syndecan-4 siRNA for 48 h, followed by incubation with SalB or TM stimulation. Immunoblot was performed to determine Syndecan-4, Rac1, and p-ATF2 protein amounts. Cell viability was evaluated by the MTS assay (*n* = 4), and ROS amounts were determined by DCF formation based on the above description (*n* = 5). (g) BM-EPCs underwent incubation with or without Syndecan-4 siRNA for 48 h, with subsequent SalB treatment and TM stimulation, and immunoblot was carried out to assess the indicated proteins. (h) BM-EPCs underwent transfection with Rac1 or scrambled siRNA for 48 h, and mRNA and protein amounts were assessed for determining silencing efficiency. (i, j) BM-EPCs underwent incubation with or without Rac1 siRNA for 48 h, followed by treatment with SalB and TM administration. Cleaved caspase-4, cytochrome *c*, Bax, and Bcl-xL proteins were detected by immunoblot. (k, l) BM-EPCs underwent incubation with or without Rac1 siRNA for 48 h, followed by treatment with SalB (or not) and TM stimulation. Cell viability was evaluated by the MTS assay (*n* = 6), and ROS amounts were quantitated as described above (*n* = 5). Data are mean ± SD, ^∗^*P* < 0.05, ^∗∗^*P* < 0.01 versus the indicated group. ns: not significant.

Syndencan-4 represents an essential factor in intracellular signal transduction during oxidative stress. In the current assay conditions, TM administration upregulated Syndencan-4, which was starkly suppressed by SalB preincubation (Figures 5(a) and 5(b)). Notably, ER stress caused starkly elevated Rac1 and ATF2 phosphorylation levels, and this effect was suppressed by SalB (Figure 5(b)). We therefore examined whether Syndencan-4 inhibition by siRNA transfection abolishes Rac1 expression and ATF2 phosphorylation induced by TM. After Syndecan-4 silencing, RT-PCR and immunoblot were carried out to confirm knockdown (Figure 5(c)). As shown in Figure 5(d), upregulated Syndecan-4, Rac1, and p-ATF2 by TM treatment were significantly attenuated when cells were silenced for Syndecan-4. Furthermore, Syndecan-4 knockdown markedly decreased TM-induced cell injury, and blunted SalB-related protection, as determined in cell viability and ROS assessment assays (Figures 5(e) and 5(f)). TM-induced CHOP and GRP78 expression and caspase-4 and caspase-3 cleavage were substantially suppressed upon Syndecan-4 silencing in comparison with the scrambled siRNA group (Figure 5(g)), suggesting that inactivation of Syndecan-4 signaling may contribute to SalB-related protection from TM-induced ER stress in BM-EPCs.

Next, to determine whether the potential downstream protein Rac1 modulates SalB-related protection, Rac1 was silenced by the siRNA technology (Figure 5(h)). As shown in Figures 5(i) and 5(j), TM-induced the activation of cytochrome *c*, and the cleavage of caspase-4 was markedly inhibited, while the increase in the Bax/Bcl − xL ratio triggered by TM was prevented after Rac1 silencing. In addition, Rac1 silencing reduced ROS amounts (70.5% compared to the nonsilenced TM group, *P* < 0.01) and increased cell viability (1.49-fold compared to the nonsilenced TM group, *P* < 0.01) after TM-associated ER stress in BM-EPCs (Figures 5(k) and 5(l)), indicating Rac1 loss protected from TM-associated damage. More importantly, SalB-related protection was markedly blunted after Rac1 silencing in BM-EPCs.

### 3.6. SalB Mitigates TM-Induced NLRP3 Inflammasome Assembly and Pyroptosis in BM-EPCs

**Figure 6:**
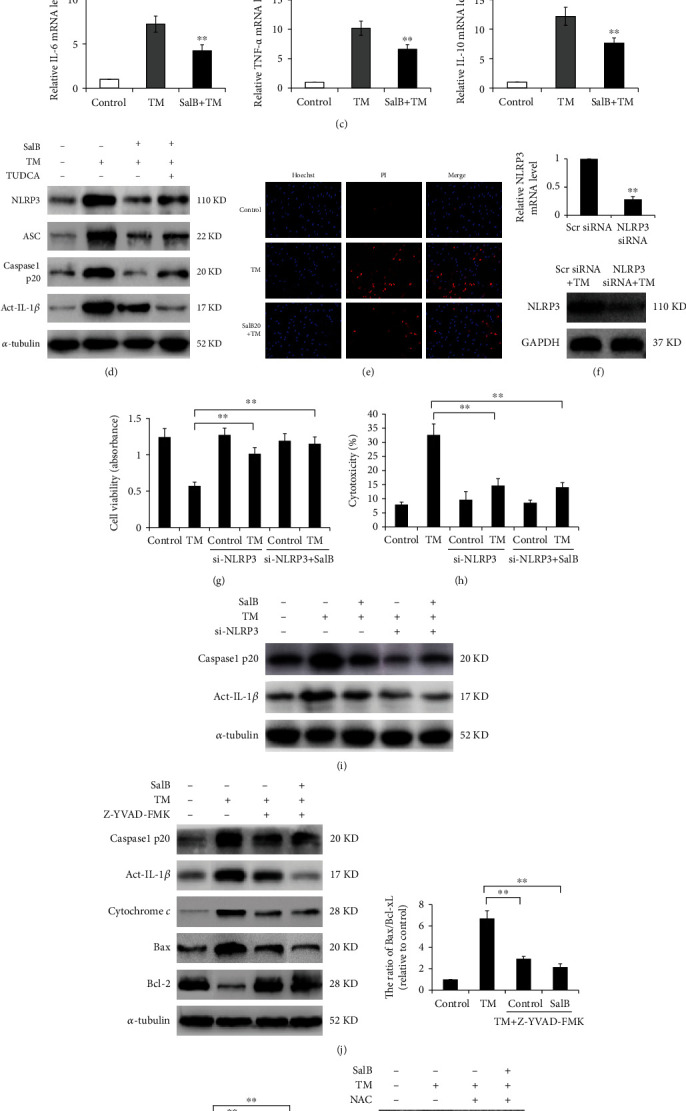
SalB mitigates TM-induced NLRP3 inflammasome assembly and pyroptosis in BM-EPCs. (a–c) BM-EPCs underwent incubation with 0 or 20 *μ*M SalB for 48 h and exposure to TM at 5 *μ*g/mL for 24 h. Cell culture supernatants were obtained following 48 h of treatment, and secreted IL-1*β* and IL-18 amounts were examined by ELISA. NLRP3, IL-1*β*, IL-18, IL-1*α*, TNF-*α*, vWF, and IL-10 mRNA amounts were determined by qRT-PCR. The 2^−*ΔΔ*Ct^ method was utilized for analysis, with GAPDH as the reference gene (*n* = 5). (d) Cells were treated with 0 or 20 *μ*M SalB for 48 h and administered TM at 5 *μ*g/mL for 24 h, and cell lysates were examined by immunoblot. (e) Cells underwent treatment with 0 or 20 *μ*M SalB for 48 h and exposure to TM at 5 *μ*g/mL; after Hoechst 33342 counterstaining, pyroptotic cells were labeled with PI. (f) BM-EPCs underwent transfection with NLRP3 or scrambled siRNA for 48 h, and mRNA and protein amounts were assessed for determining silencing efficiency. (g–i) BM-EPCs underwent incubation with or without NLRP3 siRNA for 48 h, with subsequent SalB treatment and TM stimulation; cell viability and cell death were evaluated by the MTS and LDH assays, respectively, and protein amounts were quantitated by immunoblot. (j, k) Following 48 h incubation with 20 *μ*M SalB, cells underwent treatment with the caspase-3 inhibitor z-YVAD-FMK (20 *μ*M) for 90 min before TM administration. Western blot and LDH assay (*n* = 5) were subsequently performed. (l) BM-EPCs underwent pretreatment with SalB or an ROS inhibitor (NAC), with subsequent stimulation with TM for 24 h, and immunoblot was carried out. Data are mean ± SD, ^∗^*P* < 0.05, ^∗∗^*P* < 0.01 versus the indicated group.

Under ROS conditions, TXNIP is dissociated from thioredoxin to bind to NLRP3, inducing the inflammasome to regulate inflammation [23]. Therefore, we assessed pyroptosis in BM-EPCs that could be mediated by the NLRP3 inflammasome resulting from TM exposure. Since caspase-1 cleavage and IL-*β* maturation are important markers of NLRP3 inflammasome induction, cleaved caspase-1 (Casp1 p20) and mature IL-1*β* amounts were assessed as a surrogate of NLRP3 activation [24]. As shown in Figures 6(a)–6(c), NLRP3 expression was activated in BM-EPCs stimulated by TM. After recruitment by the NLRP3 complex, caspase-1 undergoes cleavage into its p20 active form, which was starkly upregulated upon TM treatment. In this study, secreted IL-1*β* and IL-18 amounts and higher mRNA levels were detected in response to TM treatment, and these effects were mitigated by SalB (Figures 6(b) and 6(c)). In addition, we elucidated that SalB also reduced the mRNA amounts of other cytokines, including IL-6, IL-10, and TNF-*α*, in response to TM treatment in BM-EPCs (Figure 6(c)). However, the ER stress inhibitor TUDCA markedly repressed NLRP3 inflammasome and IL-1*β* activation, as well as IL-1*β* expression (Figure 6(d)).

Since NLRP3 induction results in pyroptosis, cell damage was assessed by cell viability and cytotoxicity assays. As shown in Figure 6(e), TM markedly increased PI uptake in BM-EPCs, which was restrained by SalB pretreatment. Furthermore, NLRP3 was silenced to explore its role in pyroptotic cell death mediated by ER stress (Figure 6(f)). As shown in Figures 6(g) and 6(h), NLRP3 knockdown elevated cell viability and reduced LDH release during ER stress, confirming that NLRP3 contributes to ER stress-associated pyroptosis. Additionally, NLRP3 silencing decreased SalB's effects on caspase-1 activation and mature IL-1*β* levels in ER stress-induced EPCs, suggesting the NLRP3 inflammasome is important in caspase-1 induction and pyroptosis occurrence in the current cell model (Figure 6(i)).

To further assess if TM-associated pyroptosis in BM-EPCs requires caspase-1 activation, caspase-1 inhibition assays were carried out. We found that Z-YVAD-FMK pretreatment blunted caspase-1 activation, ensuing IL-1*β* maturation upon TM exposure (Figure 6(j)). Pyroptotic cell death was reversed by Z-YVAD-FMK, as reflected by reduced LDH release (Figure 6(k)). Additionally, NLRP3, cleaved caspase-1 (Casp1 p20), and mature IL-1*β* protein amounts were all reduced by NAC (Figure 6(l)), indicating that ROS production orchestrated the exaggerated NLRP3 inflammasome activation in BM-EPCs.

Taken together, these results suggested that BM-EPCs might undergo pyroptosis induced by the NLRP3 inflammasome under the control of the mitochondrial intrinsic pathway following TM administration, and SalB might exert its protective effect on endothelial function by inhibiting NLRP3 inflammasome-dependent pyroptosis.

### 3.7. Modulation of AMPK/FoxO4/KLF2 and Syndecan-4/Rac1/ATF-2 Signaling by SalB Attenuates TM-Mediated Cell Pyroptosis

**Figure 7:**
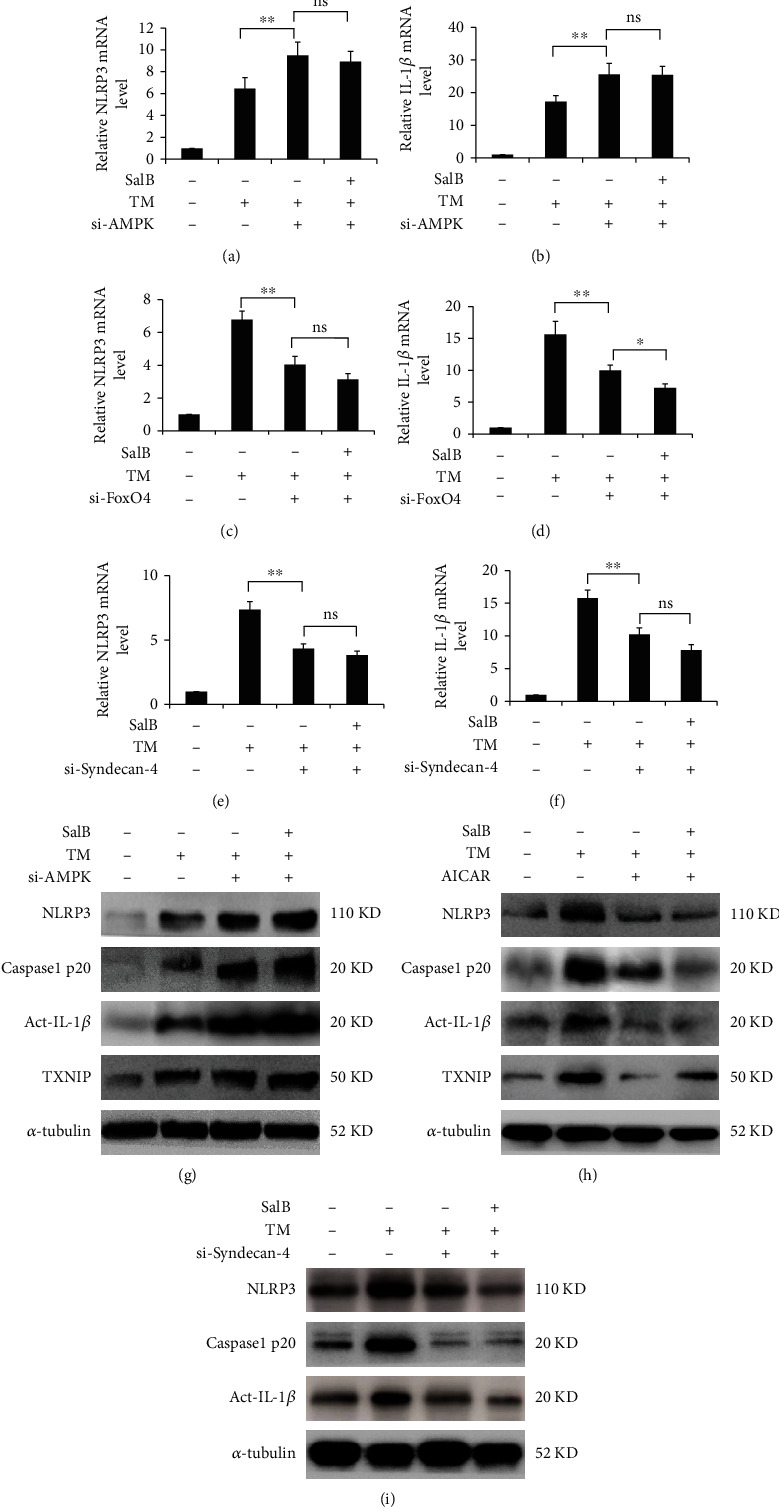
Modulation of AMPK/FoxO4/KLF2 and Syndecan-4/Rac1/ATF-2 signaling by SalB attenuates TM-mediated cell pyroptosis. (a–f) BM-EPCs underwent incubation with or without AMPK, FoxO4, or Syndecan-4 siRNA for 48 h, followed by treatment with SalB and TM stimulation. Cell culture supernatants were obtained following 48 h of treatment, and the gene expression levels of NLRP3 and IL-1*β* were determined by qRT-PCR. The 2^−*ΔΔ*Ct^ method (*n* = 5) was utilized for analysis, with GAPDH as a reference gene. (g) BM-EPCs underwent incubation with or without AMPK siRNA for 48 h, followed by treatment with SalB and TM stimulation. Cell lysates were assessed by immunoblot. (h) Cells underwent incubation with SalB (20 *μ*M) or AICAR (500 *μ*M) in the presence of TM for 24 h. Cell lysates were assessed by immunoblot. (i) BM-EPCs underwent incubation with or without Syndecan-4 siRNA for 48 h, followed by treatment with SalB and TM stimulation. Indicated proteins were detected by immunoblot. Data are mean ± SD, ^∗^*P* < 0.05, ^∗∗^*P* < 0.01 versus the indicated group. ns: not significant.

To further examine the involvement of AMPK and Syndecan-4 signaling in cell pyroptosis, cells were knocked down by siRNA transfection, and the consequence in terms of inflammasome activation was investigated. Compared with the scrambled siRNA group, AMPK knockdown dramatically decreased NLRP3 and IL-1*β* mRNA and protein amounts and blunted the effect of SalB (Figures 7(a), 7(b), and 7(g)). In addition, caspase-1 secretion was significantly promoted by AMPK silencing (Figure 7(g)). Given the critical function of TXNIP in connecting ER stress and inflammation, TM and AMPK knockdown were examined for their regulatory effects on TXNIP in BM-EPCs. TM administration upregulated TXNIP, a change reversed after AMPK silencing, indicating that SalB suppressed TXNIP induction via positive modulation of AMPK activity (Figure 7(g)). However, the AMPK activator AICAR markedly suppressed NLRP3 inflammasome components and diminished the suppressive effects of SalB, indicating the potential involvement of AMPK (Figure 7(h)). Additionally, cells with low expression of FoxO4 had decreased mRNA amounts of NLRP3 and IL-1*β* compared with those treated with TM alone (Figures 7(c) and 7(d)). Moreover, knockdown of Syndecan-4 inhibited NLRP3 inflammasome activation in BM-EPCs and alleviated SalB-associated attenuation of on NLRP3/procaspase-1 assembly in response to TM (Figures 7(e), 7(f), and 7(i)).

## 4. Discussion

ER stress is involved in impaired endothelial function. Although oxidative stress, inflammatory response, and apoptotic cell death are all found in endothelial dysfunction, ER stress has emerged as a possible cause. Aberrant and prolonged ER stress contributes to multiple pathologies, since it could cause inflammation and cytotoxicity in cells and tissues. SalB attracts mounting attention as a potent antioxidant with remarkable preventive effects in multiple oxidative stress-related pathologies [25, 26]. It is admitted that SalB protects from doxorubicin-related cardiac dysfunction by suppressing ER stress as well as cardiomyocyte apoptosis through TRPC3 and TRPC6 inhibition [18]. In our previously published paper, we demonstrated that SalB significantly promotes migration and capillary tube generation in BM-EPCs, and MKK3/6-p38 MAPK-ATF2 and ERK1/2 pathway inhibition by SalB markedly prevents oxidative stress-related cell damage in BM-EPCs [27]. The present work firstly demonstrated SalB had significant cardioprotective properties in ER stress-induced damage and apoptosis in vascular progenitor cells. ER stress-related apoptosis might be caused by the upregulation of proapoptotic factors or increased ROS amounts due to ER stress. However, how ER stress induces apoptosis remains essentially undefined. It was reported that the proapoptotic transcription factor CHOP via concurrent induction of three ER transmembrane receptors, including PERK, ATF6, and IRE1, is involved in ER stress-related apoptosis [28]. As shown above, TM induced apoptosis and ER stress in BM-EPCs, and preincubation with SalB resulted in marked reductions in TM-related apoptosis and the expression of ER stress-associated proteins such as CHOP, IRE1*α*, ATF4, and eIF2*α*. TUDCA treatment or CHOP silencing effectively ameliorated ER stress and blunted the protective effect of SalB. Moreover, besides cleaving caspase-8 and caspase-3, TM cleaved and activated the ER stress-associated caspase-4. Meanwhile, caspase-4 silencing suppressed TM-related cell damage and attenuated protection by SalB. The above findings indicate ER stress might contribute to TM-associated apoptosis in BM-EPCs.

ROS accumulation is tightly associated with ER stress, both as a consequence and a regulatory factor. Indeed, increased protein folding promotes ROS accumulation, while mitochondrial ROS further induces calcium release from the ER, causing ER stress [29]. Previous findings indicate the MMP is important in the maintenance of mitochondrial function [30]. Therefore, we examined the impact of SalB on mitochondrial function and found that it effectively reduced mitochondrial ROS levels and restored the loss of MMP, demonstrating its beneficial effect on mitochondrial function during ER stress.

HO-1 has been shown to counteract the damaging effect of a variety of cellular stressors. ER stress, like oxidative stress, in multiple cell types, upregulates HO-1, which acts autocrinally to suppress ER stress-associated apoptosis [31–33]. HO-1 overexpression by a specific inducer prevents ER stress-associated apoptosis by downregulating CHOP [34]. Driven by these considerations, we demonstrated that SalB was capable of reducing ROS generation by upregulating endothelial HO-1, as well as the primary superoxide anion scavenger SOD2. In this context, we assume that SalB inhibits ER stress-associated ROS accumulation, which, as a negative feedback mechanism, prevents further ER stress exacerbation.

It was reported that abnormal ER stress contributes to multiple pathologies for its potential to induce inflammatory response and cytotoxicity. It was demonstrated that ER stress-associated inflammation is controlled by NLRP3 inflammasome activation, which requires IRE1 and PERK [15]. Pyroptosis, a highly controlled cell death, features the induction of pathways that activate NLRs, particularly the NLRP3 inflammasome and its downstream effectors IL-1*β* and IL-18. Although NLRP3 inflammasome activation is known to be associated with the pathogeneses of metabolic diseases, its pathogenetic roles in CDVs and endothelial dysfunction are incompletely understood. It was reported that inhibition of pyroptosis by pharmacological or genetic interventions has cardioprotective effects in multiple ailments [35], providing a potential tool for treating cardiovascular pathologies. Activated endothelial NLRP3 inflammasome may represent a critical inducing mechanism by which endothelial damage under atherogenic stimuli promotes vascular inflammation and noncanonical deleterious effects on endothelial and other cells [36]. In the present study, NLRP3 inflammasome-related proteins, including NLRP3, ASC, and caspase-1, were upregulated by TM, alongside enhanced LDH release and PI uptake and reduced cell viability, suggesting NLRP3 inflammasome-associated pyroptosis in BM-EPCs is triggered by ER stress. SalB suppressed NLRP3 and caspase-1 activation, thereby reducing IL-1*β* and IL-18 amounts during ER stress. The proinflammatory cytokine IL-6 represents an important downstream target of IL-1*β*; as expected, IL-6 amounts were also decreased by SalB. These results indicated that the suppressive effect of SalB on the NLRP3 inflammasome is involved in inflammation suppression. Moreover, ER stress-associated pyroptosis requires NLRP3 inflammasome activation, as demonstrated by NLRP3 silencing. Furthermore, the ER stress inhibitor TUDCA attenuated the inhibitory effect of SalB on NLRP3 inflammasome production, suggesting that SalB's inhibitory effect on NLRP3 may be due to its upstream suppression of ER stress.

Extensive evidence suggests that AMPK constitutes an energy sensor that regulates metabolic/energy homeostasis [37, 38]. In addition, AMPK is a major modulator of vascular homeostasis with pronounced proangiogenic features [39, 40]. AMPK activation in bone marrow-derived cells enhances cell proliferation and tube generation [41]. Activated AMPK increases PPAR*δ* phosphorylation and hence suppresses ER stress in high-fat-associated obesity and hypertension [42, 43]. FoxO4 was shown to be highly expressed in EPCs, and its physiological significance in the vasculature was underscored by data showing that elevated expression of FoxO4 induces the expression of Bim, which leads to increased apoptosis in EPCs [44]. Scientific evidence has also supported that upregulation of KLF2 by AMPK activation promotes human endothelial colony-forming cells by differentiation [45]. As shown above, suppression of AMPK remarkably restored the phosphorylation of FoxO4 and suppressed KLF2 expression, suggesting AMPK is important in the regulation of FoxO4 and KLF2. Importantly, SalB dramatically suppressed the impaired effect of ER stress on migration and capillary tube generation in BM-EPCs. Mechanistically, blockade of AMPK or FoxO4 significantly prevented or enforced SalB-mediated migratory and angiogenic capacities, illustrating the central role of AMPK/FoxO4 signaling in the protection of endothelial function.

To further reveal the involvement of AMPK signaling in SalB-associated ER stress and pyroptosis, we demonstrated that the inhibitory effect exerted by SalB on ER stress was reversed by AMPK or FoxO4 silencing. In addition, knockdown of AMPK or FoxO4 blunted SalB's antipyroptotic effect, with altered NLRP3 amounts and cell damage, suggesting that AMPK/FoxO4 prevents pyroptosis via differential control of NLRP3 inflammasome amounts or activity. ROS signaling is known to contribute to NLRP3 inflammasome priming and activation [46]. Mitochondrial damage could cause the release of oxidized mtDNA into the cytoplasm, which interacts with and activates the NLRP3 inflammasome [47]. TXNIP represents an important signaling node linking ER stress and inflammation. Although it was recently demonstrated endothelial inflammation and cell death require TXNIP/NLRP3 inflammasome activation in rats [48], how ER stress and NLRP3 inflammasome activation are related in endothelial cells remains undefined. Here, AMPK/FoxO4 pathway blockade in TM-treated cells markedly reversed ROS levels modulated by SalB, illustrating that ER stress and the ROS pathway may jointly act for initiating endothelial injury. Moreover, ROS was required for ER stress-associated NLRP3 assembly as suppressing mitochondrial ROS release by the ROS scavenger NAC abrogated NLRP3-dependent pyroptosis in EPCs in response to ER stress. Moreover, corroborating a study demonstrating that TXNIP is indispensable in NLRP3 inflammasome activation in endothelial cells, this study showed that TXNIP upregulation linked ER stress to activated NLRP3 inflammasome in endothelial cells. Thus, it is tempting to speculate that pharmacological activation of AMPK by SalB inhibits TXNIP/NLRP3 inflammasome activation during ER stress, thereby resulting in a significant amelioration in mitochondrial function and a decrease in pyroptosis. Jointly, the above findings confirmed SalB protects BM-EPCs from TM-related ER stress by inhibiting NLRP3 inflammasome activation via AMPK/FoxO4 signaling.

Syndecan-4, a heparan sulfate proteoglycan, has high expression in endothelial and epithelial cells in multiple organs [49]. Syndecan-4 can cooperate with many receptors to subsequently play regulatory roles in different processes, including wound healing, inflammation, and angiogenesis [49, 50]. In addition, Syndecan-4-mediated Rac1 activation is important in ROS production and MAPK/JNK activation [51, 52]. It was also reported that pharmacological inhibition of Rac1 improves endothelial function and attenuates atherosclerosis development in mice [53]. Exploring the mechanism underpinning SalB-related cytoprotection, we found that SalB treatment significantly reduced the expression of Syndecan-4/Rac1/ATF2. Blockade of Syndecan-4 using siRNA effectively reversed ER stress-related CHOP and GRP78 activation, as well as caspase-4 cleavage. Additionally, blockade of Rac1 increases cell survival as reflected by elevated resistance to ER stress. Corroborating its effect on cell viability, Syndecan-4 silencing attenuated pyroptotic cell death via an NLRP3 inflammasome-based mechanism, suggesting that Syndecan-4 activation might be responsible for ER stress-induced pyroptosis in BM-EPCs. In addition, we present evidence supporting that Syndecan-4 silencing abolishes the attenuating effect of SalB on pyroptosis under ER stress, suggesting an important role for Syndecan-4/Rac1 signaling in SalB-associated cytoprotection.

## 5. Conclusions

Overall, this study demonstrated that due to the regulation of AMPK/FoxO4/KLF2 and Syndecan-4/Rac1/ATF2 signaling, SalB suppresses ER stress, downregulates TXNIP, and suppresses NLRP3 inflammasome activation by downregulating NLRP3 and cleaved caspase-1. This leads to decreased IL-18 and IL-1*β* secretion amounts, ultimately attenuating ER stress-induced endothelial injury. The current findings support a new role for SalB in decreasing NLRP3 inflammasome activation and subsequent pyroptosis, demonstrating SalB's potential as an agent for treating atherosclerosis.

## Data Availability

The data used to support the findings of this study are available from the corresponding authors upon request.

## Abbreviations

AS: Atherosclerosis
BM-EPC: Bone marrow-derived endothelial progenitor cell
SalB: Salvianolic acid B
ER: Endoplasmic reticulum
TM: Tunicamycin
ROS: Reactive oxygen species
DCF: Dichlorodihydro-fluorescein
MMP: Mitochondrial membrane potential
NAC: N-acetyl-l-cysteine
TUDCA: Tauroursodeoxycholic acid
AICAR: 5-Aminoimidazole-4-carboxamide 1-*β*-D-ribofuranoside
FBS: Fetal bovine serum
CHOP: C/EBP-homologous protein
GRP78: 78 kDa glucose-regulating protein
ATF4: Activating transcription factor 4
eIF2*α*: Eukaryotic initiation factor 2
HO-1: Heme oxygenase-1
SOD2: Superoxide dismutase 2
NLRP3: NOD-like receptor family of protein 3
TXNIP: Thioredoxin-interacting protein
ATF2: Activating transcription factor 2
AMPK: AMP-activated protein kinase
FoxO4: Forkhead box O4
KLF2: Kruppel-like factor
Rac1: Ras-related C3 botulinum toxin substrate 1
IL-1*β*: Interleukin-1*β*
IL-18: Interleukin-18
TNF-*α*: Tumor necrosis factor-*α*.

## References

[1] Coffeng J. K., van der Ploeg H. P., Castellano J. M. (2017). A 30-month worksite-based lifestyle program to promote cardiovascular health in middle-aged bank employees: design of the TANSNIP-PESA randomized controlled trial. *American Heart Journal*.

[2] Ray S., Miglio C., Eden T., del Rio D. (2014). Assessment of vascular and endothelial dysfunction in nutritional studies. *Nutrition, Metabolism, and Cardiovascular Diseases*.

[3] Leuti A., Fazio D., Fava M., Piccoli A., Oddi S., Maccarrone M. (2020). Bioactive lipids, inflammation and chronic diseases. *Advanced Drug Delivery Reviews*.

[4] Lorenzatti A. J., Servato M. L. (2019). New evidence on the role of inflammation in CVD risk. *Current Opinion in Cardiology*.

[5] Hersh D., Monack D. M., Smith M. R., Ghori N., Falkow S., Zychlinsky A. (1999). The Salmonella invasin Sip B induces macrophage apoptosis by binding to caspase-1. *Proceedings of the National Academy of Sciences of the United States of America*.

[6] Franchi L., Munoz-Planillo R., Nunez G. (2012). Sensing and reacting to microbes through the inflammasomes. *Nature Immunology*.

[7] Duewell P., Kono H., Rayner K. J. (2010). NLRP3 inflammasomes are required for atherogenesis and activated by cholesterol crystals. *Nature*.

[8] He Y., Hara H., Nunez G. (2016). Mechanism and regulation of NLRP3 inflammasome activation. *Trends in Biochemical Sciences*.

[9] He B., Nie Q., Wang F. (2021). Role of pyroptosis in atherosclerosis and its therapeutic implications. *Journal of Cellular Physiology*.

[10] Ochoa C. D., Wu R. F., Terada L. S. (2018). ROS signaling and ER stress in cardiovascular disease. *Molecular Aspects of Medicine*.

[11] Ghemrawi R., Battaglia-Hsu S. F., Arnold C. (2018). Endoplasmic reticulum stress in metabolic disorders. *Cell*.

[12] Reverendo M., Mendes A., Argüello R. J., Gatti E., Pierre P. (2019). At the crossway of ER-stress and proinflammatory responses. *The FEBS Journal*.

[13] Cnop M., Toivonen S., Igoillo-Esteve M., Salpea P. (2017). Endoplasmic reticulum stress and eIF2*α* phosphorylation: the Achilles heel of pancreatic *β* cells. *Molecular Metabolism*.

[14] Chen X., Guo X., Ge Q., Zhao Y., Mu H., Zhang J. (2019). ER stress activates the NLRP3 inflammasome: a novel mechanism of atherosclerosis. *Oxidative Medicine and Cellular Longevity*.

[15] Oslowski C. M., Hara T., O'Sullivan-Murphy B. (2012). Thioredoxin-interacting protein mediates ER stress-induced *β* cell death through initiation of the inflammasome. *Cell Metabolism*.

[16] Tait S. W., Ichim G., Green D. R. (2014). Die another way--non-apoptotic mechanisms of cell death. *Journal of Cell Science*.

[17] Wang Y., Xu F., Chen J. (2011). Matrix metalloproteinase-9 induces cardiac fibroblast migration, collagen and cytokine secretion: inhibition by salvianolic acid B from Salvia miltiorrhiza. *Phytomedicine*.

[18] Chen R. C., Sun G. B., Ye J. X., Wang J., Zhang M. D., Sun X. B. (2017). Salvianolic acid B attenuates doxorubicin-induced ER stress by inhibiting TRPC3 and TRPC6 mediated Ca^2+^ overload in rat cardiomyocytes. *Toxicology Letters*.

[19] Tang Y., Huang B., Sun L., Peng X., Chen X., Zou X. (2011). Ginkgolide B promotes proliferation and functional activities of bone marrow-derived endothelial progenitor cells: involvement of Akt/eNOS and MAPK/p38 signaling pathways. *European Cells & Materials*.

[20] Zhou R., Yazdi A. S., Menu P., Tschopp J. (2011). A role for mitochondria in NLRP3 inflammasome activation. *Nature*.

[21] Fujiki T., Ando F., Murakami K. (2019). Tolvaptan activates the Nrf2/HO-1 antioxidant pathway through PERK phosphorylation. *Scientific Reports*.

[22] Zhu X., Wang K., Zhou F., Zhu L. (2018). Paeoniflorin attenuates atRAL-induced oxidative stress, mitochondrial dysfunction and endoplasmic reticulum stress in retinal pigment epithelial cells via triggering Ca(2+)/CaMKII-dependent activation of AMPK. *Archives of Pharmacal Research*.

[23] Han Y. C., Xu X. X., Tang C. Y. (2018). Reactive oxygen species promote tubular injury in diabetic nephropathy: the role of the mitochondrial ros-txnip-nlrp3 biological axis. *Redox Biology*.

[24] Takahashi M. (2014). NLRP3 inflammasome as a novel player in myocardial infarction. *International Heart Journal*.

[25] Ji Q. Q., Li Y. J., Wang Y. H. (2020). Salvianolic acid B improves postresuscitation myocardial and cerebral outcomes in a murine model of cardiac arrest: involvement of Nrf2 signaling pathway. *Oxidative Medicine and Cellular Longevity*.

[26] Xiao Z., Liu W., Mu Y. P. (2020). Pharmacological effects of salvianolic acid B against oxidative damage. *Frontiers in Pharmacology*.

[27] Tang Y. B., Jacobi A., Vater C., Zou X., Stiehler M. (2014). Salvianolic acid B protects human endothelial progenitor cells against oxidative stress-mediated dysfunction by modulating Akt/mTOR/4EBP1, p38 MAPK/ATF2, and ERK1/2 signaling pathways. *Biochemical Pharmacology*.

[28] Sano R., Reed J. C. (2013). ER stress-induced cell death mechanisms. *Biochimica et Biophysica Acta*.

[29] Zhang K., Kaufman R. J. (2008). From endoplasmic-reticulum stress to the inflammatory response. *Nature*.

[30] Geldon S., Fernandez-Vizarra E., Tokatlidis K. (2021). Redox-mediated regulation of mitochondrial biogenesis, dynamics, and respiratory chain assembly in yeast and human cells. *Frontiers in Cell and Development Biology*.

[31] Khakurel A., Park P. H. (2018). Globular adiponectin protects hepatocytes from tunicamycin-induced cell death via modulation of the inflammasome and heme oxygenase-1 induction. *Pharmacological Research*.

[32] Liang Y., Fan C., Yan X. (2019). Berberine ameliorates lipopolysaccharide-induced acute lung injury via the PERK-mediated Nrf2/HO-1 signaling axis. *Phytotherapy Research*.

[33] Maamoun H., Benameur T., Pintus G., Munusamy S., Agouni A. (2019). Crosstalk between oxidative stress and endoplasmic reticulum (ER) stress in endothelial dysfunction and aberrant angiogenesis associated with diabetes: a focus on the protective roles of heme oxygenase (HO)-1. *Frontiers in Physiology*.

[34] Liu X. M., Peyton K. J., Ensenat D. (2005). Endoplasmic reticulum stress stimulates heme oxygenase-1 gene expression in vascular smooth muscle. Role in cell survival. *The Journal of Biological Chemistry*.

[35] Zhang Y., Liu X., Bai X. (2018). Melatonin prevents endothelial cell pyroptosis via regulation of long noncoding RNA MEG3/miR-223/NLRP3 axis. *Journal of Pineal Research*.

[36] Boini K. M., Hussain T., Li P. L., Koka S. S. (2018). Trimethylamine-N-oxide instigates NLRP3 inflammasome activation and endothelial dysfunction. *Cellular Physiology and Biochemistry: International Journal of Experimental Cellular Physiology, Biochemistry, and Pharmacology*.

[37] Jiang T., Yu J. T., Zhu X. C. (2015). Ischemic preconditioning provides neuroprotection by induction of AMP-activated protein kinase-dependent autophagy in a rat model of ischemic stroke. *Molecular Neurobiology*.

[38] Rabinovitch R. C., Samborska B., Faubert B. (2017). AMPK maintains cellular metabolic homeostasis through regulation of mitochondrial reactive oxygen species. *Cell Reports*.

[39] Jansen T., Kröller-Schön S., Schönfelder T. (2018). alpha1AMPK deletion in myelomonocytic cells induces a pro-inflammatory phenotype and enhances angiotensin II-induced vascular dysfunction. *Cardiovascular Research*.

[40] Spengler K., Zibrova D., Woods A. (2020). Protein kinase a negatively regulates VEGF-induced AMPK activation by phosphorylating CaMKK2 at serine 495. *The Biochemical Journal*.

[41] Zhu Z., Fu C., Li X. (2011). Prostaglandin E2 promotes endothelial differentiation from bone marrow-derived cells through AMPK activation. *PloS One*.

[42] Liu L. M., Liu J., Huang Z. X. (2015). Berberine improves endothelial function by inhibiting endoplasmic reticulum stress in the carotid arteries of spontaneously hypertensive rats. *Biochemical and Biophysical Research Communications*.

[43] Cheang W. S., Tian X. Y., Wong W. T. (2014). Metformin protects endothelial function in diet-induced obese mice by inhibition of endoplasmic reticulum stress through 5′ adenosine monophosphate-activated protein kinase-peroxisome proliferator-activated receptor *δ* pathway. *Arteriosclerosis, Thrombosis, and Vascular Biology*.

[44] Urbich C., Knau A., Fichtlscherer S. (2005). FOXO-dependent expression of the proapoptotic protein Bim: pivotal role for apoptosis signaling in endothelial progenitor cells. *The FASEB Journal*.

[45] Song Y. M., Li X. X., Wang D. W. (2013). Transcription factor Krüppel-like factor 2 plays a vital role in endothelial colony forming cells differentiation. *Cardiovascular Research*.

[46] Tschopp J., Schroder K. (2010). NLRP3 inflammasome activation: the convergence of multiple signalling pathways on ROS production?. *Nature Reviews Immunology*.

[47] Shimada K., Crother T. R., Karlin J. (2012). Oxidized mitochondrial DNA activates the NLRP3 inflammasome during apoptosis. *Immunity*.

[48] Mohamed I. N., Hafez S. S., Fairaq A., Ergul A., Imig J. D., el-Remessy A. B. (2014). Thioredoxin-interacting protein is required for endothelial NLRP3 inflammasome activation and cell death in a rat model of high-fat diet. *Diabetologia*.

[49] Vuong T. T., Reine T. M., Sudworth A., Jenssen T. G., Kolset S. O. (2015). Syndecan-4 is a major Syndecan in primary human endothelial cells in vitro, modulated by inflammatory stimuli and involved in wound healing. *The Journal of Histochemistry and Cytochemistry*.

[50] Xie J., Wang J., Li R. (2012). Syndecan-4 over-expression preserves cardiac function in a rat model of myocardial infarction. *Journal of Molecular and Cellular Cardiology*.

[51] Kim J., Lee J. H., Park H. S. (2008). Syndecan-4 regulates platelet-derived growth factor-mediated MAP kinase activation by altering intracellular reactive oxygen species. *FEBS Letters*.

[52] Elfenbein A., Simons M. (2013). Syndecan-4 signaling at a glance. *Journal of Cell Science*.

[53] Zimmer S., Goody P. R., Oelze M. (2021). Inhibition of Rac1 GTPase decreases vascular oxidative stress, improves endothelial function, and attenuates atherosclerosis development in mice. *Frontiers in Cardiovascular Medicine*.

